# Exploitation of Lactic Acid Bacteria and Baker’s Yeast as Single or Multiple Starter Cultures of Wheat Flour Dough Enriched with Soy Flour

**DOI:** 10.3390/biom10050778

**Published:** 2020-05-18

**Authors:** Bernadette-Emőke Teleky, Adrian Gheorghe Martău, Floricuța Ranga, Felicia Chețan, Dan C. Vodnar

**Affiliations:** 1 Institute of Life Sciences, University of Agricultural Sciences and Veterinary Medicine, Calea Mănăștur 3-5, 400372 Cluj-Napoca, Romania; bernadette.teleky@usamvcluj.ro (B.-E.T.); adrian.martau@usamvcluj.ro (A.G.M.); 2 Faculty of Food Science and Technology, University of Agricultural Sciences and Veterinary Medicine, Calea Mănăștur 3-5, 400372 Cluj-Napoca, Romania; floricutza_ro@yahoo.com; 3 Agricultural Research and Development Station Turda, str. Agriculturii, nr. 27, Turda, 401100 Jud. Cluj, Romania; felice_fely@yahoo.com

**Keywords:** lactic acid bacteria, soybean, sourdough, fermentation, *Saccharomyces*, organic acids, viscoelastic behavior

## Abstract

Sourdough fermentation presents several advantageous effects in bread making, like improved nutritional quality and increased shelf life. Three types of experiments aimed to evaluate comparatively the efficiency of two *Lactobacillus* (Lb.) strains, *Lb. plantarum* ATCC 8014 and *Lb. casei* ATCC 393, to metabolize different white wheat flour and soybeans flour combinations to compare their efficiency, together with/without *Saccharomyces cerevisiae* on sourdough fermentation. For this purpose, the viability, pH, organic acids, and secondary metabolites production were investigated, together with the dynamic rheological properties of the sourdough. During sourdough fermentation, LAB presented higher growth, and the pH decreased significantly from above pH 6 at 0 h to values under 4 at 24 h for each experiment. Co-cultures of LAB and yeast produced a higher quantity of lactic acid than single cultures, especially in sourdough enriched with soy-flour. In general, sourdoughs displayed a stable, elastic-like behavior, and the incorporation of soy-flour conferred higher elasticity in comparison with sourdoughs without soy-flour. The higher elasticity of sourdoughs enriched with soy-flour can be attributed to the fact that through frozen storage, soy proteins have better water holding capacity. In conclusion, sourdough supplemented with 10% soy-flour had better rheological properties, increased lactic, acetic, and citric acid production.

## 1. Introduction

Sourdough used in the food industry, is a traditional leaven agent, for the fermentation of dough with microorganisms like yeasts and lactic acid bacteria (LAB) [1,2]. The use of sourdough fermentation in bread leavening is gaining increased attention as it is recognized as a healthy and natural way of bread making [3]. With a history of over 5000 years, sourdough fermentation presents several beneficial effects like a heterogeneous and enhanced sensory quality of baked foods [4,5]. Furthermore, with the improvement of its nutritional quality, sourdough has the added benefit of increasing bread shelf life, which brings the added advantage of diminished or no use of preservatives [6].

White wheat flour is an essential staple food worldwide and contributes to the everyday intake of daily fiber, micronutrient, and energy, but it presents fewer nutrients in comparison to whole-wheat flour [7,8]. In addition, soybeans contain a high content of isoflavones (daidzein, glycitein, and genistein) that have beneficial health effects like osteoporosis and cardiovascular disease prevention, anti-tumor agent, and hinders Alzheimer disease [9,10]. Soy flour addition to wheat flour contributes to the nutritional characteristic of wheat flour dough. To increase bread quality, several studies analyze the beneficial effect of soy flour addition to wheat flour [11,12,13]. On the other hand, soy flour supplementation due to the high content of soy proteins (45–55 g/100 g of soy flour) is feasible only in low quantity otherwise, loaf volume is diminished, has inferior crumb properties, and reduced acceptance [11,14]. The health-promoting effects of soy proteins are highly studied [15,16], although in small children, it is believed to be a major allergen [17]. Aguirre et al. [18] observed the ability of different LAB strains to degrade the main soy proteins, until 6 h of fermentation, especially β-conglicinin. Seeing that LAB hydrolyzes primary soy proteins, they can be efficiently used for better digestion of soy proteins permitting their incorporation in an everyday diet [18]. A recent study [19] analyzed the effect of soy flour addition on bread dough through frozen storage, which had a positive effect on dough extensibility strength and hardness. Textural and sensorial data indicated that qualitatively soy dough has a slower deterioration rate in comparison with wheat dough. A 48.5% of soy-flour addition acted as a plasticizing agent due to effective water-binding capability and provided nutritional advantages [19].

**Figure 1:**
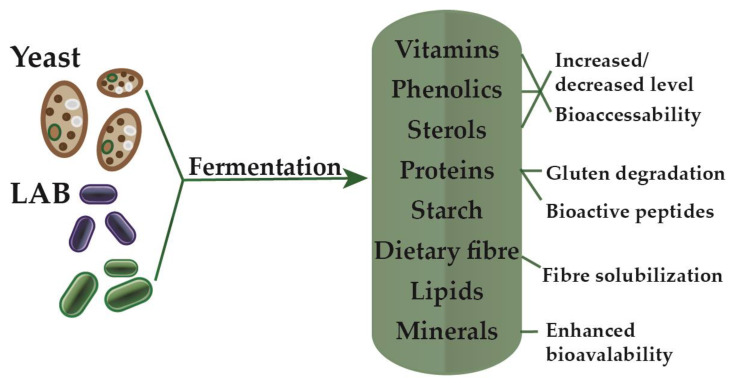
Nutritional influence of sourdough fermentation with yeast and lactic acid bacteria (LAB).

During cereal fermentation, typically up to 24 h at moderate temperatures, the metabolic activity of the microorganisms present is in interaction with the grain constituents. In general, LAB produce lactic and acetic acids, lowering the pH typically below pH 5. Additionally, usually, yeasts produce carbon dioxide and ethanol. Interactions between yeasts and bacteria are essential for the metabolic activity of the sourdough, such as the bioconversion of complex carbohydrates into organic acids or other chemical compounds with bioactive potential [20]. The changing conditions during fermentation contribute to the activation of enzymes present, and adjustment of pH selectively enhances the performance of certain enzymes, such as amylase, proteases, hemicellulases, and phytases [21]. The enzyme-induced changes, together with microbial metabolites, bring about the technological and nutritional effects of fermented cereal foods. Sourdough fermentation can influence the nutritional quality by decreasing or increasing levels of compounds and enhancing or retarding the bioavailability of nutrients (Figure 1) [22]. An important aspect of sourdough is regarding to starch digestibility [23]. Owing to the high digestibility of gelatinized starch and as in wheat bread starch is in a strongly gelatinized and porous form, this aspect leads to the fast growth of blood glucose level [22,24,25]. Reduced digestibility is an advantageous effect of sourdough fermentation because of organic acid production, like lactic acid that decreases the digestion of starch [22,24].

In functional food production, the use of LAB is favored due to its unique characteristics. LAB is competent in producing polyols, antimicrobial substances, nutraceuticals, valuable enzymes, or aromatic compounds [26,27,28]. LAB consists of Gram-positive bacteria with corresponding physiological, metabolic, and morphological features, and can transform sugars to lactic acid [29].

During food fermentations, often-encountered microorganisms are LAB and yeasts. *Saccharomyces cerevisiae* (*Sc*), a commercial baker’s yeast, remains the usual selection in baking. *Sc* has fast growth, through dough leavening, great biomass yield on molasses medium, and presents increased CO_2_ production [30]. Supported by its capacity to ferment carbohydrates *Lactobacillus plantarum* (*Lp*), a heterofermentative LAB is one of the native dominant strains of sourdough [31]. *Lp* ferments hexoses through the Embden-Meyerhof Parnas pathway [32] and is extensively used in processing and fermentation of unprocessed foods (vegetable, meat, and dairy). This LAB is “generally recognized as safe” (GRAS) with a status of qualified presumption of safety (QPS-EFSA, 2018) [29,33]. The facultative heterofermentative *Lactobacillus casei (Lc)* ferments nearly all hexose sugars to lactic acid [34]. *Lc* is a highly used LAB in food fermentation for flavor enhancement in the cheese industry and in sourdough production to manipulate acidification, gluten protein deterioration, aroma compounds, and amino acid production and several other nutritional and sensory improvement [35]. Utilization of complex microbial consortia is the general method used for performing food fermentation techniques, but there are also several studies analyzing single cultures [36,37,38]. According to recent studies, there are reciprocally stimulating interactions between LAB and *Sc.* Yeasts are a crucial element in foodstuff and beverage production [2], and are predominantly in charge of dough leavening [39].

The antifungal activity of *L. plantarum* 21B was also found in sourdough bread. Compared to bread started with *S. cerevisiae* 141 alone or in association with *L. brevis* 1D, the sourdough bread, which used *L. plantarum* 21 B in association with *S. cerevisiae* 141 delayed fungal contamination until after seven days of storage at room temperature [40].

The food industry to be efficient and competing has to address consumer requirements and recent trends, which have incorporated the need for high-quality foods that are not highly processed and do not involve any chemical preservatives. Considering the antimicrobial compounds generated by LAB are regarded as natural preservatives, the use of *L. plantarum* 21 B to decrease the fungal contamination of sourdough baked products has interesting potential applications [40].

The objectives of the present study were to (1) characterize the outcome of soy flour addition to wheat flour during fermentation; (2) analyze the conversion level of wheat flour and soy flour through fermentation with two heterofermentative lactic acid bacteria (LAB) strains *Lactobacillus plantarum* (*Lp*) and *Lactobacillus casei* (*Lc*) as single strain starter cultures, and in multiple strain starters together with baker’s yeast *Saccharomyces cerevisiae* (*Sc*); and (3) to characterize the dynamic rheological properties and effect of soy flour addition on bread dough throughout a 24 h fermentation after frozen storage.

## 2. Materials and Methods

### 2.1. Materials

Culture media components and other reagents were of analytical grade and obtained from VWR International (Radnor, Pennsylvania, PA, USA) except peptone special obtained from Sigma-Aldrich (Steinheim, Germany), and agar (Agar plant for cell culture) obtained from Applichem (Omaha, NE, USA).

### 2.2. Microorganisms and Culture Conditions

The microorganisms used throughout this study were two *Lactobacillus* (*Lb*.) strains, *Lb. plantarum* ATCC 8014 and *Lb. casei* ATCC 393, obtained from the University of Agricultural Science, and Veterinary Medicine Cluj-Napoca and *Sc* (Pak Gida Uretim ve Pazarlama AS, Turkey) acquired from commerce. The medium used for LAB was MRS broth (per liter: glucose, 20.0 g; yeast extract, 5.0 g; meat extract, 10.0 g; enzymatic digest of casein, 10.0 g; sodium acetate, 5.0 g; diammonium citrate, 2.0 g; dipotassium hydrogen phosphate, 2.0 g; magnesium sulphate, 0.2 g; manganese sulphate, 0.05 g; and polyoxyethylene sorbitan monooleate, and 1.08 g with a final pH of 6.4 ± 0.2 at 25 °C). For the *Sc* yeast strain, the medium used was GPY (per liter: glucose, 40.0 g; peptone, 5.0 g; and yeast extract, 5.0 g).

Microorganisms’ reactivation before experimental usage was in 9 mL MRS media by introducing 1 mL of LAB inoculum or 1 g of dried yeast in GPY media. Vial incubation performed at 30 °C (yeast) and 37 °C (LAB) for 18–24 h. The second propagation occurred in MRS/GPY broth, through the inoculation of the activated LAB or yeast (10 mL) and afterward incubated for 18–24 h.

**Figure 2:**
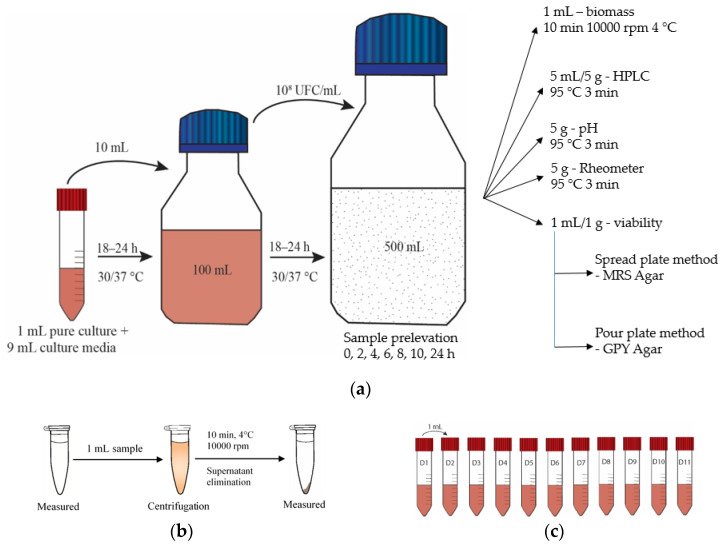
(**a**). Microorganism activation, (**b**) washing process, and (**c**) dilutions.

Microorganism concentration of 10^8^ CFU/mL was determined with the spectrophotometer NanoDrop 1000 (NanoDrop Technologies, Wilmington, DE, USA) through optical density measurement at 600 nm (OD600) between values 0.009 and 0.011 for bacteria or with Thoma counting chamber (Marienfeld, Germany) under the microscope (Nikon, Japan) for yeasts [41]. In 500 mL model media (MRS/GPY) the experiment started, following the addition of 50 mL from the established concentration of 10^8^ bacteria/yeast. Samples for HPLC (5 mL), viability (1 mL), and wet biomass (1 mL) extracted at 0, 2, 4, 6, 8, 10, 12 h, and at 24 h to monitor the changes (Figure 2). Two single and one co-culture fermentation: (S1) *Lp*, (S2) *Lc*, (S3) *Lp*, *Lc*, and *Sc*.

### 2.3. Sourdough Preparation

A commercial wheat flour, used for traditional bread making (type 000, according to the ash content by the Romanian classification), with 15.3% moisture and 11.2% protein, was used. The sourdough preparation included ratio flour: water of 1:1, to produce a dough yield of 200 (dough mass/flour mass × 100). The soybean provided by the Agricultural Research and Development Center Turda (https://scdaturda.ro/onix/). The obtained soybean variety was Onix (*Glycine max* (L.) Merril), with conventional soil cultivation system through tillage and 60% autumn rape vegetable debris (green fertilizer). Soybeans were ground (SF) and added to the wheat flour in the amount of 5% and 10%. The preparation consisted of three types of fermentation with 100% WF (batch A), 95% WF with 5% SF (batch B), and 90% WF with 10% of soy flour addition (batch C). Before fermentation, the measured wheat quantities went through a sterilization process, and after the addition of 100 mL of autoclaved deionized water, the dough went through a homogenization step. The ratio of obtained sourdough was 1 g^−1^ mL, with a final yield of 200 mL.

### 2.4. Fermentations

The fermentation of the different wheat concentrations with simple and co-cultures were carried out separately. Sample prelevation as described before, as with the simple culture media, the only difference was that for viability, samples were prelevated with sterile sample spoons and weighing boats. For viability with pour plate method [42] first, 12-fold, respectively, 9-fold serial dilutions were made in tubes with 9 mL physiological saline solution and 1 mL broth. In the Petri dish, 1 mL of diluted inoculum and about 15 mL of warm MRS agar poured and mixed after which it was left to solidify. Fungi viability developed with a spread plate method on potato dextrose agar. On the solidified agar, 100 µL inoculum spread evenly with a glass Drigalsky spatula on the surface of the agar. Plates for LAB were incubated at 37 °C for 48 h and for fungi at 25 °C for 48–72 h.

Before inoculation in wheat and soy flour broth, the culture media was centrifuged 10 min at 4 °C, 7000 rpm, the supernatant discarded, and the pellet suspended with saline solution [43]. After this washing step repeated two times, with NanoDrop, LAB concentration was measured, and the fungi were counted with Thoma Counting Chamber.

### 2.5. Organic Acid and Secondary Metabolite Analysis by HPLC

After fermentation extraction and quantification of organic acids and secondary metabolites was possible with the help of high-performance liquid chromatography (HPLC-Agilent 1200 series, Santa Clara, CA, USA) equipped with solvent degasser, quaternary pumps, DAD detector coupled with a mass detector, column thermostat, and automatic injector (Agilent Technologies, Santa Clara, CA, USA). The separation of organic acids could be realized on reversed-phase chromatographic column Acclaim OA (5 µm, 4 mm × 150 mm Dionex), eluted for 10 min with monosodium phosphate solution (NaH_2_PO_4_) 50 mM concentration, pH 2.8, and a flow rate of 0.5 mL/min, at a temperature of 20 °C. The measurement of chromatograms was possible at the wavelength λ = 210 nm.

Sample preparation for the HPLC consisted of the addition of 2 mL distilled H_2_O to 1 g of sample, which was vortexed 30 sec, sonicated 15 min, and centrifuged at 8000 rpm for 10 min at 4 °C. After these steps, the supernatant was filtered with a Millipore membrane filter of 0.45 µm pore size [44]. A volume of 20 µL of sample was injected in the column, with a flow rate of 0.5 mL/min, and detection conducted at 280 and 340 nm [45].

### 2.6. pH Measurements

The pH measurement through the in vitro experiments was determined with a digital pH meter (InoLab 7110, Germany) at room temperature through dissolving 5.0 g of sample in 50 mL of double-distilled water [46].

### 2.7. Rheological Measurements 

For rheological measurements, samples were kept at −20 °C (frozen storage), and before measurements, they were defrosted at room temperature. The dough’s dynamic rheological characteristics were analyzed utilizing an Anton Paar MCR 72 rheometer (Anton Paar, Graz, Austria) [47,48], supplied with a Peltier plate-plate system (P-PTD 200/Air) with temperature control and a 50 mm diameter smooth parallel plate geometry (PP-50-67300). On the lower plate, after supplying around 3 g of dough, the upper plate was lowered to a plate distance set at a gap of 1 mm. After the removal of the dough surplus from the exterior of the upper plate geometry, to avoid sample drying through testing, silicone oil was added.

### 2.8. Statistical Analysis

The results of three independent assays (performed with replicates each) were expressed as mean value ± SD, *n* = 3. The statistical evaluation was carried out using Graph Prism Version 8.0.1. (GraphPad Software Inc., San Diego, CA, USA) through a one-way ANOVA (Tukey multiple comparisons tests). Differences among means at a 5% level have been considered statistically significant.

## 3. Results and Discussions

### 3.1. pH and Cell Viability

In general, for all fermentations, reached cell counts for LAB were of 10^10^–10^12^ CFU/mL for model media and 10^8^–10^9^ CFU/mL for sourdough fermentation (Table 1). These cell counts were reached during the first 24 h fermentation. Initially, the analysis of microorganism viability on model media, presented a high increase, especially the LAB *Lc* (S2) and in the co-cultures (S3) with a final concentration of above 10^12^ CFU/mL. The viability on three different substrates reached a final concentration above 10^9^ CFU/mL. The highest viability observed was with the LAB *Lp*, and the increase was uniform throughout the experiment, while *Lc* at the beginning presented a slower growth, but the final concentration was similar to *Lp.* No negative effect on the viability of LAB could be observed in the presence of *Sc*. In contrary LAB together with *Sc* yeast, presented the highest viability on substrate with 10% and 5% soy flour addition. Comparing the results in a similar study [49] LAB presented a lower initial (10^6^ CFU/mL) and final cell density (10^9^ CFU/mL at 24 h) in model media and the same results as in our study in sourdough.

Soy flour addition to wheat flour had a positive effect especially in fermentations with *Lc,* reaching final values of 5.7 × 10^9^ CFU/mL in comparison to the values obtained with 100% wheat flour of 3.3 × 10^9^ CFU/mL. In co-cultures with the addition of 10% soy flour, the viability also increased for LAB (from 1.1 × 10^9^ CFU/mL with 100% wheat flour to 2.9 × 10^9^ CFU/mL with 10% soy flour) and *Sc* (from 1.8 × 10^7^ CFU/mL with 100% wheat flour to 4.1 × 10^7^ CFU/mL with 10% soy flour). As demonstrated by Aguirre et al. [18,50] several LAB efficiently degrade soy as a substrate, especially *Lp.* Depending on the applied fermentation conditions, especially refreshment time and the substrate, the pH values differed (Table 1). The lowest pH values were obtained with co-cultures (3.4) in model media. In the case of model media, due to carbohydrates like glucose that was more accessible, the viability increased, and the pH decreased faster than sourdough where the microorganisms had to break down starch to glucose. 

At sourdough fermentation, the lowest pH value (3.4–3.6) was reached in fermentation batch C, and the highest (3.8–3.9) was reached in fermentation batch A. These data indicated the growth and metabolic activity of the LAB during the whole fermentation period.

In a study from Paucean et al. [49], pH started from approximately 6.12. After 24 h in modified model media, the pH reached a final value of 4.35 with *Lp* and 4.57 with *Lc* in media containing only glucose as a carbohydrate. Similar results were obtained with maltose (*Lp*-4.81 and *Lc*-4.62), and in media with glucose and fructose (*Lp*-4.1 and *Lc*-4.85). In media containing only fructose (*Lp*-5.59 and *Lc*-5.64), the pH had not decreased substantially. In sourdoughs, the obtained pH values reached the same results as in the present study <4.0, and the pH became stable after 20–22 h [49,51].

### 3.2. Rheological Measurements

**Figure 3:**
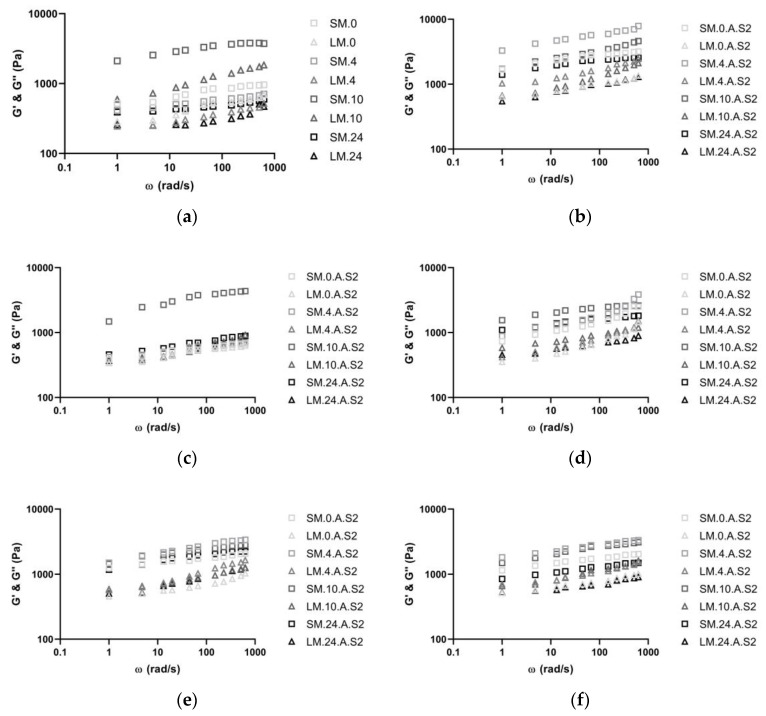

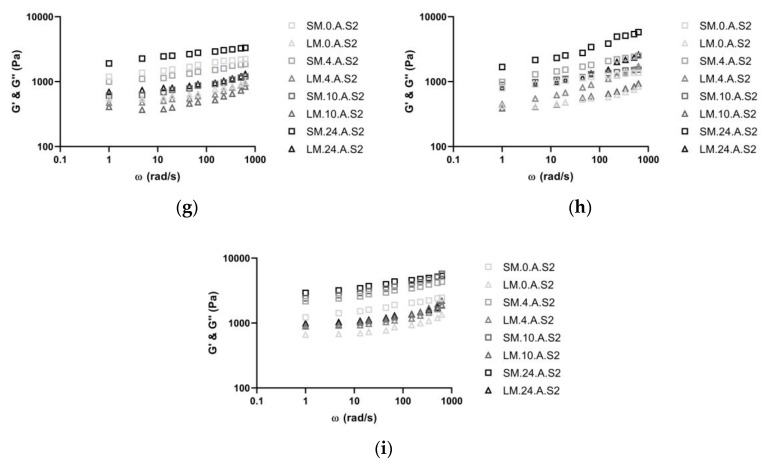
The storage (G’) and loss (G’’) shear moduli for sourdoughs fermented in 100% wheat flour with (**a**) *Lp*, (**b**) *Lc*, and (**c**) *Lp* + *Lc* + *Sc*; 95% wheat + 5% soy flour with (**d**) *Lp*, (**e**) *Lc*, and (**f**) *Lp* + *Lc* + *Sc*; and 90% wheat + 10% soy flour with (**g**) *Lp*, (**h**) *Lc*, and (**i**) *Lp* + *Lc* + *Sc* through 24 h.

Rheological property alterations of sourdoughs with/without soy-flour addition and the effect of fermentation with single and co-cultures were evaluated (Figure 3 and Supplementary Tables S1–S9). The storage modulus (G’) and loss modulus (G’’) of each sourdough at an angular frequency of 0.628–628 rad s^−1^ are presented in Figure 3a–i. The capability of materials to store the elastic deformation energy is represented by G’, while G’’ corresponds to the viscous portion of the materials [52]. It can be observed that both moduli (G’ and G’’) of every sourdough increased with the increase of angular frequency.

In general, G’ was higher than G’’, illustrating that every sourdough sample displayed a stable, elastic-like behavior. The effect of soy flour addition to wheat flour increased the elasticity of doughs in every fermentation, but especially with the *Lc* microorganism. At a final angular frequency of 628 rad s^−1^ at 24 h the loss modulus increased with *Lp* from 599.1 ± 6.1 to 3313.6 ± 6.7, with *Lc* from 2581.3 ± 11.1 to 5790.3 ± 5.8 with 10%, and in co-cultures from 889.5 ± 3.9 with no soy flour to 5326 ± 10.1 with 10% of soy flour addition.

In batch A and B, G’ and G’’ decreased from 10 to 24 h of fermentation and in batch C at 24 h G’ and G’’ were higher than at the beginning of the fermentation. The higher elasticity in batch C can be explained mainly by the water holding capacity of soy proteins during frozen storage (−20 °C) and the disruption of sourdough macromolecules like gluten proteins [19]. Soy and wheat proteins are capable of binding covalently and non-covalently (e.g., disulfide and hydrogen bonds) [53], and the strong binding capacity of soy protein to water is less affected through freezing [53].

Wheat flour combined with water results in a hydrated gluten network that gives the viscoelastic property of sourdough. The right amount of water is important in sourdough preparation, and due to non-miscible properties of wheat biopolymers, it is present in each phase of bread making [54]. The study of sourdough rheological properties is associated with different wheat or other types of flours and their water absorption capacity [55]. Sun et al. [56] modeled the viscoelastic behavior of a broad range of doughs prepared in different formulations. According to this study, with the increase of water content, the viscosity decreases. Hardt et al. [55] found that the reduction of water content from 43.5% to 34% caused a positive growth in dough consistency, and a similar result was obtained with the increase of xylanase addition.

The effect of fermentation with different *Lp* strains proved that through fermentation of whole wheat bread, the quality and shelf life of bread could be improved. In this study, with the use of *Lp* LB-1 stronger extensibility, increased viscoelasticity, and water retention ability was obtained.

The production of organic acids and the decrease of pH influence the rheological properties, and reduce mixing time, which causes an increased elastic behavior [57]. Soybean flour addition increased the overall organic acid production, with each single or co-culture, and, as a consequence, influenced positively the elastic behavior of sourdough. Zhou et al. [58] reported enhanced stability and elasticity of wheat dough enriched with soy-protein that provided an increased water-binding ability. As stated, soy-protein strengthens the disulphide linkage, offering better elasticity for baked products.

### 3.3. Organic Acid and Secondary Metabolite Analysis by HPLC

LAB greatly influences the sensory, textural, nutritional, and shelf-life characteristics of sourdough baked goods, especially bread [59]. For a long time, the improved shelf life of sourdough baked products was attributed to the lactic and acetic acids produced by LAB [60,61]. Further studies [62,63,64] have shown that lactic acid is not inhibitory to fungi, while the acetic acid concentration seems to be more strictly related to the antifungal activity [40]. Acetic acid through sourdough fermentation also enhances the aroma profile of bread [65]. The acetic acid concentration in sourdough may be increased by adding fructose, which is used as an external electron acceptor by heterofermentative LAB, which consequently increases the growth yield and acetic acid production [40].

In the present study, the ratio of acetic acid production in model media was elevated, but in sourdough was very low (Table 2, Table 3 and Table 4) or there was no production at all, which is in concordance with similar studies [49,51]. However, where there was a 10% soy flour addition (Table 4) it can be seen that after 24 h of fermentation there was a slight acetic acid production of 0.022 ± 0.03 g/L with *Lp,* 0.186 ± 0.07 g/L with *Lc,* and the highest production was observed with co-cultures of 0.294 ± 0.10 g/L.

Depending on the used LAB through the sourdough fermentation taking place, the metabolism of organic acids like citrate, fumarate, and malate. Lactate, malate, and citrate transformation uses intracellular protons and consequently enhances the LAB’s tolerance to the acidic environment [32]. In the present study, fermentation most probably uses the pathway of converting citrate into lactate (no acetic acid production in our case) together with malate fermentation.

Lactic acid concentration at 0 h in sourdoughs was absent or in a very low concentration (0.3–0.5 g/L). After a fermentation of 24 h, the highest lactic acid production could be observed with *Lp*, especially in sourdoughs from batch B with 5% soy flour. The production of lactic acid in batch B with single *Lp* culture was 3.787 ± 0.03 g/L and in co-cultures together with *Lc* and *Sc* was 4.879 ± 0.03 g/L. The lowest lactic acid production was observed in batch A with both single cultures (2.137–2.178 g/L), but with co-cultures lactic acid production increased significantly to 3.787 g/L.

Fumaric acid presented a low but continuous amount during the whole 24 h of fermentation. According to several studies, malic and fumaric acid are usually converted to succinic acid in LAB [66,67,68].

The production of malic acid, primarily used in the food industry as a taste intensifier and an acidulant is especially a result of yeast fermentation [69]. Malic acid production in these experiments was in accordance with similar studies [51], which shows the equilibrium during hydrolysis of starch, which is conditioned of microbial and enzymatic transformation (amylases and maltose metabolic enzymes) at every stage of dough fermentation [70]. The presence of soy flour in fermentation increased the malic acid concentration (0.847 ± 0.39) in comparison with samples where only wheat flour was used (0.550 ± 0.14).

When LAB remains without any carbohydrate, they might utilize citric acid as an energy supply [66]. In the present study citric acid, production during fermentation presented fluctuating results, but usually, until the end of fermentation, it began to decrease. Hu et al. [71], characterized the antimicrobial activity of three *Lp* strains from isolated dairy food and proved that organic acids present an important factor as antimicrobial substances in fermentation broths. These organic acids display the best antimicrobial activity, especially if they are mixed in different concentrations. Beside lactic and acetic acid, citric, malic, and tartaric acid also possess antimicrobial activity.

Succinic acid production in model media was in concordance with Kaneuchi et al. [66], who showed that *Lactobacillus* strains produce various amounts of succinic acid in MRS media. In the present study, succinic acid production increased for *Lp* at 4 h of 9.496 ± 0.10 g/L, *Lc* at 10 h of 9.244 ± 0.09 g/L, and in co-culture at 4 h of 9.681 ± 0.11 g/L, after which it decreased. In sourdoughs, succinic acid was only produced where *Lp* was present, with an increase after 4 h of fermentation and decreased until 24 h. Succinic acid production increased with the increase of soybean flour with the highest values in batch A of 1.077 ± 0.08, batch B of 1.918 ± 0.03, and batch C of 3.993 ± 0.05 g/L. Considering *Lp,* has an incomplete tricarboxylic acid cycle and a natural producer of succinic acid; this LAB is extensively researched in studies of metabolic engineering for higher succinic acid production [72].

## 4. Conclusions

Sourdough fermentation with single and co-cultures of LAB and yeast has several beneficial effects, and soy-flour incorporation further improved sourdough quality. The addition of soy-flour to wheat flour had a positive impact, considering the fermentations with *Lc* (5.7 × 10 ^9^ CFU/mL), and co-cultures (LAB: 2.9 × 10^9^; *Sc*: 4.1 × 10 ^7^). The metabolic activity and growth of microorganisms was also observed with the decrease of pH value with final values of 3.4–3.6 with 10% of soy flour and 3.8–3.9 where no soy flour was added.

Some beneficial effects of soy-flour addition were the increase of organic acids after 24 h of fermentation production especially with *Lp* (lactic: 3.330 ± 0.01 g/L, malic: 1.004 ± 0.07 g/L, and succinic acid: 1.070 ± 0.06 g/L) and co-cultures of *Lp + Lc + Sc* (lactic: 3.580 ± 0.01 g/L and succinic acid: 0.799 ± 0.10 g/L). Acetic acid production in sourdoughs was only observed in co-cultures and also increased with the increase of soy-flour at a final value of 0.294 ± 0.10 g/L.

The incorporation of soy-flour in wheat flour during sourdough preparation generated relatively important alterations in comparison with sourdough without any soy-flour addition. The addition of 10% of soy-flour presented enhanced rheological properties, like increased elastic behavior. Although every sourdough exhibited a constant elastic-like behavior, the incorporation of soy-flour presented higher elasticity, which is due to the water retention ability of soy-protein in the course of frozen storage. The highest elasticity was obtained after 24 h of fermentation and especially where *Lc* was present.

## Figures and Tables

**Table 1:** Viability and pH of the sourdough fermentations.

Sourdough Fermentation Batch		M.M.			A		B		C	
		pH range	C.c. (CFU/g)	W.B. (g/L)	pH range	C.c. (CFU/g)	pH range	C.c. (CFU/g)	pH range	C.c. (CFU/g)
S1	Initial	6.39 ± 0.12	1 × 10^8^	0.0036 ± 0.001	6.12 ± 0.19	3.0 × 10^6^	6.07 ± 0.22	2.0 × 10^6^	6.02 ± 0.09	2.2 × 10^6^
	Final	3.58 ± 0.23	4.3 × 10^10^	0.0172 ± 0.004	3.79 ± 0.16	4.8 × 10^9^	3.81 ± 0.25	3.3 × 10^9^	3.64 ± 0.27	4.1 × 10^9^
S2	Initial	6.41 ± 0.34	3.5 × 10^8^	0.0054 ± 0.002	6.15 ± 0.21	3.0 × 10^5^	5.62 ± 0.17	8.0 × 10^5^	5.73 ± 0.35	2.0 × 10^5^
	Final	3.56 ± 0.19	9.5 × 10^12^	0.0159 ± 0.007	3.81 ± 0.08	1.5 × 10^9^	3.34 ± 0.10	3.5 × 10^9^	3.35 ± 0.15	5.7 × 10^9^
S3	Initial LAB	6.37 ± 0.12	2.0 × 10^8^	0.0069 ± 0.001	6.19 ± 0.09	3.0 × 10^5^	5.14 ± 0.05	1.5 × 10^5^	5.09 ± 0.12	3.0 × 10^5^
	Final LAB	3.36 ± 0.32	1.5 × 10^12^	0.0156 ± 0.003	3.92 ± 0.11	1.1 × 10^9^	3.55 ± 0.30	4.2 × 10^9^	3.50 ± 0.26	2.9 × 10^9^
	Initial *Sc*	6.37 ± 0.12	4.7 × 10^6^	0.0069 ± 0.001	6.19 ± 0.09	1.7 × 10^3^	5.14 ± 0.05	2.4 × 10^3^	5.09 ± 0.12	1.9 × 10^3^
	Final *Sc*	3.36 ± 0.32	1.8 × 10^8^	0.0156 ± 0.003	3.92 ± 0.11	1.8 × 10^7^	3.55 ± 0.30	2.6 × 10^7^	3.50 ± 0.26	4.1 × 10^7^

M.M.—model media, A—100% WF, B—95% WF + 5% SF, C—90% WF + 10% SF, S1—*L. plantarum*, S2—*L. casei*, S3—*L. plantarum + L. casei + S. cerevisiae*, LAB—*L. plantarum* + *L. casei*, Initial—analyzed at 0 h, final—analyzed at 24 h, W.B.—Wet Biomass, WF—wheat flour, SF—soy flour, C.c.—Cell count.

**Table 2:** Organic acid production through fermentation with *Lp.*

Org. Acids (g/L)		Lactic A.	Acetic A.	Malic A.	Succinic A.	Tartaric A.	Citric A.	Fumaric A.
Substr.	Time (h)							
MM	0	n.d.	0.663 ± 0.07 ^a^	1.384 ± 0.05 ^a^	6.609 ± 0.14 ^a^	0.342 ± 0.06 ^a^	3.081 ± 0.07 ^a^	0.033 ± 0.07 ^a^
	4	1.812 ± 0.04 ^a^	1.812 ± 0.09 ^a^	2.139 ± 0.06 ^a^	9.496 ± 0.10 ^a^	0.668 ± 0.07 ^a^	3.430 ± 0.08 ^a^	0.015 ± 0.02 ^a^
	10	2.899 ± 0.06 ^a^	2.408 ± 0.10 ^a^	1.641 ± 0.04 ^a^	8.259 ± 0.08 ^a^	0.478 ± 0.08 ^a^	1.630 ± 0.08 ^a^	0.017 ± 0.01 ^a^
	24	7.856 ± 0.06 ^a^	2.899 ± 0.09 ^a^	1.557 ± 0.09 ^a^	8.361 ± 0.11 ^a^	0.451 ± 0.12 ^a^	0.813 ± 0.05 ^a^	0.017 ± 0.02 ^a^
A	0	0.363 ± 0.08 ^a^	n.d.	0.482 ± 0.06 ^b^	0.673 ± 0.08 ^c^	0.152 ± 0.05 ^b^	n.d.	0.027 ± 0.06 ^a^
	4	0.462 ± 0.07 ^b^	n.d.	0.494 ± 0.07 ^c^	1.077 ± 0.08 ^d^	0.169 ± 0.07 ^b^	n.d.	0.029 ± 0.04 ^a^
	10	0.643 ± 0.08 ^c^	n.d.	0.570 ± 0.06 ^b^	0.572 ± 0.04 ^d^	0.186 ± 0.02 ^b^	n.d.	0.013 ± 0.04 ^a^
	24	1.514 ± 0.06 ^d^	n.d.	0.468 ± 0.10 ^b^	0.130 ± 0.03 ^d^	0.100 ± 0.08 ^b^	0.212 ± 0.03 ^b^	0.008 ± 0.02 ^a^
B	0	n.d.	n.d.	0.612 ± 0.09 ^b^	1.286 ± 0.04 ^b^	0.354 ± 0.06 ^a^	0.414 ± 0.05 ^b^	0.037 ± 0.08 ^a^
	4	n.d.	n.d.	0.619 ± 0.11 ^b,c^	1.437 ± 0.09 ^c^	0.339 ± 0.06 ^ab^	0.371 ± 0.05 ^c^	0.037 ± 0.07 ^a^
	10	1.328 ± 0.06 ^b,c^	n.d.	1.323 ± 0.04 ^a^	1.918 ± 0.03 ^c^	0.459 ± 0.04 ^a^	0.532 ± 0.08 ^b^	0.034 ± 0.06 ^a^
	24	3.787 ± 0.03 ^b^	n.d.	1.025 ± 0.09 ^a^	0.785 ± 0.08 ^c^	0.205 ± 0.07 ^b^	0.295 ± 0.07 ^b^	0.007 ± 0.01 ^a^
C	0	n.d.	n.d.	0.457 ± 0.13 ^b^	0.531 ± 0.07 ^c^	0.181 ± 0.07 ^b^	0.450 ± 0.06 ^b^	0.027 ± 0.05 ^a^
	4	n.d.	n.d.	1.471 ± 0.08 ^b^	3.993 ± 0.05 ^b^	0.589 ± 0.08 ^a^	0.979 ± 0.06 ^b^	0.024 ± 0.03 ^a^
	10	1.634 ± 0.12 ^b^	n.d.	1.393 ± 0.03 ^a^	3.564 ± 0.08 ^b^	0.598 ± 0.07 ^a^	0.648 ± 0.08 ^b^	0.010 ± 0.02 ^a^
	24	3.330 ± 0.01 ^c^	n.d.	1.004 ± 0.07 ^a^	1.070 ± 0.06 ^b^	0.393 ± 0.13 ^a^	0.362 ± 0.09 ^b^	0.001 ± 0.00 ^a^

Results (displayed as mean values ± SD, g/L, *n* = 3), in every column significant differences (*p* < 0.05) are shown with different letters (a–d) between the types of substrate used (one-way ANOVA, multiple comparisons test, and Tukey multiple range test (*p* = 0.05), GraphPad Prism, Version 8.0.1, Graph Pad S., Inc., San Diego, CA, USA). A—100% WF, B—95% WF + 5% SF, C—90% WF + 10% SF, WF—wheat flour, SF—soy flour.

**Table 3:** Organic acid production through fermentation with *Lc.*

Org. Acids (g/L)		Lactic A.	Acetic A.	Malic A.	Succinic A.	Tartaric A.	Citric A.	Fumaric A.
Substr.	Time (h)							
MM	0	2.496 ± 0.04 ^a^	3.571 ± 0.11 ^a^	1.225 ± 0.04 ^a^	3.661 ± 0.11 ^a^	0.371 ± 0.07 ^a^	2.930 ± 0.04 ^a^	0.029 ± 0.05 ^a^
	4	3.139 ± 0.08 ^a^	4.081 ± 0.09 ^a^	1.367 ± 0.14 ^a^	8.448 ± 0.09 ^a^	0.463 ± 0.08 ^a^	3.141 ± 0.07 ^a^	0.020 ± 0.09 ^a^
	10	4.972 ± 0.10 ^a^	3.062 ± 0.06 ^a^	1.831 ± 0.05 ^a^	9.244 ± 0.09 ^a^	0.558 ± 0.06 ^a^	3.174 ± 0.07 ^a^	0.014 ± 0.07 ^a^
	24	8.329 ± 0.04 ^a^	2.369 ± 0.12 ^a^	1.870 ± 0.08 ^a^	3.787 ± 0.08 ^a^	0.495 ± 0.05 ^a^	1.091 ± 0.07 ^a^	0.015 ± 0.04 ^a^
A	0	n.d.	n.d.	0.402 ± 0.06 ^b^	n.d.	0.099 ± 0.03 ^b^	0.225 ± 0.06 ^c^	0.023 ± 0.01 ^a^
	4	n.d.	n.d.	0.518 ± 0.11 ^b^	n.d.	0.325 ± 0.06 ^a^	1.052 ± 0.10 ^b^	0.024 ± 0.12 ^a^
	10	0.931 ± 0.12 ^b^	n.d.	0.473 ± 0.07 ^b^	n.d.	0.297 ± 0.06 ^a,b^	0.242 ± 0.09 ^b^	0.022 ± 0.08 ^a^
	24	2.137 ± 0.01 ^b^	n.d.	0.737 ± 0.07 ^b^	n.d.	0.259 ± 0.05 ^a,b^	0.521 ± 0.10 ^b^	0.033 ± 0.06 ^a^
B	0	n.d.	n.d.	0.451 ± 0.07 ^b^	n.d.	0.217 ± 0.07 ^a,b^	0.559 ± 0.07 ^b^	0.025 ± 0.04 ^a^
	4	0.534 ± 0.07 ^b^	n.d.	0.966 ± 0.05 ^a,b^	n.d.	0.301 ± 0.09 ^a^	0.808 ± 0.08 ^b,c^	0.032 ± 0.02 ^a^
	10	0.149 ± 0.05 ^b^	n.d.	0.491 ± 0.07 ^b^	n.d.	0.303 ± 0.04 ^a,b^	0.364 ± 0.10 ^b^	0.010 ± 0.01 ^a^
	24	1.878 ± 0.04 ^c^	n.d.	0.666 ± 0.12 ^b^	n.d.	0.205 ± 0.04 ^b^	0.273 ± 0.08 ^b^	0.003 ± 0.01 ^a^
C	0	n.d.	n.d.	0.451 ± 0.06 ^b^	n.d.	0.126 ± 0.06 ^b^	0.176 ± 0.7 ^c^	0.020 ± 0.04 ^a^
	4	n.d.	n.d.	0.506 ± 0.04 ^b^	n.d.	0.200 ± 0.05 ^a^	0.427 ± 0.08 ^c^	0.031 ± 0.07 ^a^
	10	0.349 ± 0.08 ^b^	n.d.	0.432 ± 0.11 ^b^	n.d.	0.128 ± 0.02 ^b^	0.231 ± 0.10 ^b^	0.001 ± 0.00 ^a^
	24	2.212 ± 0.11 ^b^	n.d.	0.657 ± 0.05 ^b^	n.d.	0.456 ± 0.08 ^a^	1.133 ± 0.11 ^a^	0.004 ± 0.01 ^a^

Results (displayed as mean values ± SD, g/L, *n* = 3), in every column significant differences (*p* < 0.05) are shown with different letters (a–d) between the types of substrate used (one-way ANOVA, multiple comparisons test, and Tukey multiple range test (*p* = 0.05), GraphPad Prism, Version 8.0.1, Graph Pad S., Inc., San Diego, CA, USA). A—100% WF, B—95% WF + 5% SF, C—90% WF + 10% SF, WF—wheat flour, SF—soy flour.

**Table 4:** Organic acid production through fermentation with *Lp + Lc + Sc.*

Org. Acids (g/L)		Lactic A.	Acetic A.	Malic A.	Succinic A.	Tartaric A.	Citric A.	Fumaric A.
Substr.	Time (h)							
MM	0	2.079 ± 0.09 ^a^	2.870 ± 0.10 ^a^	1.501 ± 0.10 ^a^	8.057 ± 0.09 ^a^	0.407 ± 0.08 ^a^	4.604 ± 0.08 ^a^	0.016 ± 0.04 ^a^
	4	3.267 ± 0.08 ^a^	4.004 ± 0.05 ^a^	1.689 ± 0.09 ^a^	9.681 ± 0.11 ^a^	0.517 ± 0.11 ^a^	3.688 ± 0.08 ^a^	0.013 ± 0.07 ^a^
	10	4.715 ± 0.06 ^a^	4.147 ± 0.08 ^a^	1.724 ± 0.09 ^a^	7.907 ± 0.07 ^a^	0.387 ± 0.09 ^a^	1.814 ± 0.12^b^	0.021 ± 0.04 ^a^
	24	4.935 ± 0.07 ^a^	4.918 ± 0.07 ^a^	0.934 ± 0.1 ^a^	8.094 ± 0.18	0.395 ± 0.07 ^a^	1.996 ± 0.06 ^a^	0.021 ± 0.09 ^a^
A	0	n.d.	n.d.	0.359 ± 0.07 ^c^	n.d.	0.436 ± 0.04 ^a^	0.391 ± 0.05 ^c^	0.044 ± 0.02 ^a^
	4	n.d.	n.d.	0.682 ± 0.06 ^a,b^	n.d.	0.556 ± 0.10 ^a^	1.244 ± 0.07 ^b^	0.049 ± 0.07 ^a^
	10	0.578 ± 0.06 ^b^	n.d.	0.838 ± 0.03 ^b^	n.d.	0.203 ± 0.02 ^b^	0.389 ± 0.09 ^c^	0.037 ± 0.08 ^a^
	24	2.178 ± 0.03 ^c^	0.022 ± 0.03 ^d^	0.575 ± 0.11 ^b^	n.d.	0.176 ± 0.08 ^b^	0.198 ± 0.09 ^c^	0.010 ± 0.03 ^a^
B	0	n.d.	n.d.	0.675 ± 0.10 ^b^	n.d.	0.314 ± 0.11 ^a^	0.752 ± 0.13 ^b^	0.051 ± 0.10 ^a^
	4	n.d.	n.d.	0.355 ± 0.04 ^b^	n.d.	0.116 ± 0.04 ^b^	0.630 ± 0.08 ^b^	0.020 ± 0.08 ^a^
	10	0.899 ± 0.06 ^b^	n.d.	1.152 ± 0.06 ^b^	n.d.	0.252 ± 0.01 ^a,b^	0.603 ± 0.09 ^b,c^	0.030 ± 0.07 ^a^
	24	4.879 ± 0.03 ^a^	0.186 ± 0.07 ^c^	1.263 ± 0.02 ^a^	n.d.	0.334 ± 0.05 ^a^	0.396 ± 0.04 ^b,c^	0.003 ± 0.06 ^a^
C	0	0.553 ± 0.13 ^b^	n.d.	0.964 ± 0.05 ^a,b^	n.d.	0.335 ± 0.08 ^a^	0.887 ± 0.09 ^b^	0.032 ± 0.08 ^a^
	4	0.339 ± 0.07 ^b^	n.d.	1.180 ± 0.06 ^a^	n.d.	0.379 ± 0.06 ^a,b^	1.252 ± 0.08 ^b^	0.043 ± 0.03 ^a^
	10	0.921 ± 0.06 ^b^	n.d.	1.212 ± 0.08 ^b^	1.026 ± 0.07 ^b^	0.359 ± 0.05 ^a^	0.986 ± 0.07 ^b^	0.018 ± 0.08 ^a^
	24	3.580 ± 0.01 ^b^	0.294 ± 0.10 ^b^	0.533 ± 0.02 ^b^	0.799 ± 0.10 ^b^	0.375 ± 0.02 ^a^	0.633 ± 0.12 ^b^	0.003 ± 0.07 ^a^

Results (displayed as mean values ± SD, g/L, *n* = 3), in every column significant differences (*p* < 0.05) are shown with different letters (a–d) between the types of substrate used (one-way ANOVA, multiple comparisons test, and Tukey multiple range test (*p* = 0.05), GraphPad Prism, Version 8.0.1, Graph Pad S., Inc., San Diego, CA, USA). A—100% WF, B—95% WF + 5% SF, C—90% WF + 10% SF, WF—wheat flour, SF—soy flour.

## Supplementary Materials

The following are available online at https://www.mdpi.com/2218-273X/10/5/778/s1, Table S1. Standard deviation of storage (G’) and loss (G’’) shear moduli for 100% wheat flour with *Lp*; Table S2. Standard deviation of storage (G’) and loss (G’’) shear moduli for 100% wheat flour with *Lc*; Table S3. Standard deviation of storage (G’) and loss (G’’) shear moduli for 100% wheat flour with *Lp* + *Lc* + *Sc*; Table S4. Standard deviation of storage (G’) and loss (G’’) shear moduli for 95% wheat + 5% soy flour with *Lp*; Table S5. Standard deviation of storage (G’) and loss (G’’) shear moduli for 95% wheat + 5% soy flour with *Lc*; Table S6. Standard deviation of storage (G’) and loss (G’’) shear moduli for 95% wheat + 5% soy flour with *Lp* + *Lc* + *Sc*; Table S7. Standard deviation of storage (G’) and loss (G’’) shear moduli for 90% wheat + 10% soy flour with *Lp*; Table S8. Standard deviation of storage (G’) and loss (G’’) shear moduli for 90% wheat + 10% soy flour with *Lc*; Table S9. Standard deviation of storage (G’) and loss (G’’) shear moduli for 90% wheat + 10% soy flour with *Lp* + *Lc* + *Sc*.
