# Supplementary material for: Exploitation of Lactic Acid Bacteria and Baker’s Yeast as Single or Multiple Starter Cultures of Wheat Flour Dough Enriched with Soy Flour
Source: Biomolecules. 2020 May 18;10(5):778. doi: 10.3390/biom10050778 (PMC7277752; doi:10.3390/biom10050778)
Supplement: Supplementary file 1 [file biomolecules-10-00778-s001.pdf]

Supplementary Table 1. Standard deviation of storage (G′) and loss (G′′) shear moduli for 100% wheat flour with *Lp*.

	**0.628**	**5**	**13**	**20**	**45**	**68**	**151**	**227**	**341**	**513**	**628**
SM.0	517.1 ± 0.9	540.1 ± 3.6	644.5 ± 4.2	693.6 ± 8.3	812.8 ± 3.3	843.5 ± 7.1	856.1 ± 6.8	910.7 ± 8.3	936.3 ± 8.7	946.3 ± 8.1	960.6 ± 8
LM.0	284.7 ± 2.6	296.4 ± 2.2	356.8 ± 5.6	402.8 ± 5.6	504.0 ± 1.8	528.0 ± 6.7	539.5 ± 3.5	569.8 ± 6.5	597.0 ± 6.5	626.9 ± 5.6	693.9 ± 7.1
SM.4	412.3 ± 3.1	452.3 ± 6.5	501.8 ± 7.5	511.3 ± 3.5	552.8 ± 6.4	584.1 ± 5.1	609.1 ± 5.8	625.7 ± 5	649.1 ± 5.7	673.2 ± 5.1	705.9 ± 5.6
LM.4	245.5 ± 2.9	254.8 ± 1.9	278.4 ± 8.3	304.1 ± 2.3	333.5 ± 2.2	358.3 ± 2.3	391.3 ± 2.3	427.7 ± 4.3	446.2 ± 3.4	458.7 ± 3.6	517.1 ± 4.1
SM.10	2111.8 ± 9	2558 ± 12.3	2863 ± 15.1	3003 ± 10.8	3310 ± 11.3	3474 ± 16	3654 ± 13.2	3771 ± 16.5	3797 ± 14.3	3795 ± 11	3733 ± 13.2
LM.10	595.1 ± 0.8	725.9 ± 4.3	874.9 ± 7.3	952 ± 6.8	1135 ± 9.7	1266 ± 9.9	1399 ± 11.5	1559 ± 14.5	1649 ± 12.5	1738 ± 6.8	1846 ± 10.8
SM.24	391.5 ± 2.8	403.9 ± 2.3	433.7 ± 2.9	437.6 ± 5.4	461.8 ± 7.5	473.9 ± 4.4	492.6 ± 6.5	517.1 ± 6.5	539.5 ± 6.9	570.3 ± 3.1	599.1 ± 5.3
LM.24	258.8 ± 1.5	252.5 ± 1.1	259.1 ± 1.7	255.9 ± 0.9	272.4 ± 3.1	288.5 ± 1.5	314.5 ± 3.6	343.8 ± 3.2	365.7 ± 7.8	507.3 ± 2.2	470.9 ± 6.1

Results (displayed as mean values ±SD, n = 3), SM – storage modulus, LM – loss modulus.

Supplementary Table 2. Standard deviation of storage (G′) and loss (G′′) shear moduli for 100% wheat flour with *Lc***.**

	**0.628**	**5**	**13**	**20**	**45**	**68**	**151**	**227**	**341**	**513**	**628**
SM.0	1557.5 ± 11	2081 ± 10.5	2339.9 ± 8	2452 ± 11.5	2682.4 ± 11	2790.7 ± 19	2872.7 ± 9	2938.2 ± 9.7	3013.3 ± 14	3122.3 ± 11	3176 ± 9.8
LM.0	643 ± 15.2	692 ± 7.4	787.8 ± 7.3	822.5 ± 9.6	931.1 ± 12.3	1017 ± 11.2	1061 ± 11.2	1100 ± 11.7	1137.7 ± 9.7	1244.4 ± 9.1	1357.5 ± 7.6
SM.4	3291 ± 17.3	4202 ± 18.3	4702 ± 18.6	4922.1 ± 13	5432.5 ± 9.7	5703.8 ± 9.7	5936.1 ± 15.1	6491 ± 8.9	6701.1 ± 10	7008.1 ± 13	7853.4 ± 12
LM.4	1028 ± 9.8	1086 ± 9.6	1239 ± 10	1308.3 ± 9.5	1478.7 ± 8.5	1592.2 ± 5.6	1766.7 ± 14.6	2026 ± 14.5	2115.4 ± 9.9	2230.4 ± 9.9	2447 ± 11
SM.10	1713 ± 10.5	2238.6 ± 11	2538 ± 12.3	2669.6 ± 7.6	2912.4 ± 13.2	3075.3 ± 9.9	3471.7 ± 13.7	3712.2 ± 9	3984.7 ± 12	4413 ± 15.6	4610.8 ± 8.9
LM.10	666.7 ± 7	730.7 ± 8.7	879.6 ± 8.3	932.3 ± 3.8	1088.5 ± 9	1202 ± 11.7	1438.2 ± 8.9	1597.5 ± 5.8	1779.6 ± 10	1971.8 ± 14	2108.9 ± 9.3
SM.24	1406 ± 9.6	1777.5 ± 6	1958.2 ± 7	2063.6 ± 7.7	2286.2 ± 11.3	2333.7 ± 5.6	2402.1 ± 10.5	2463.4 ± 8.3	2542.4 ± 15	2568.8 ± 11	2581.3 ± 11.1
LM.24	547.3 ± 3.2	632.1 ± 2.2	771.1 ± 3.5	798.1 ± 5	925.6 ± 9.1	976.3 ± 6.4	1022 ± 11.7	1071 ± 11.2	1171.2 ± 9.7	1231.9 ± 8.6	1294.8 ± 7.8

Results (displayed as mean values ±SD, n = 3), SM – storage modulus, LM – loss modulus.

Supplementary Table 3. Standard deviation of storage (G′) and loss (G′′) shear moduli for 100% wheat flour with *Lp* + *Lc* + *Sc*.

	**0.628**	**5**	**13**	**20**	**45**	**68**	**151**	**227**	**341**	**513**	**628**
SM.0	382.9 ± 1.9	429.2 ± 1.8	488.8 ± 10.2	514.9 ± 4.8	540.8 ± 8.8	545.8 ± 5.7	585.9 ± 4.8	620.5 ± 6.7	649.2 ± 7.2	657.9 ± 6.1	678.3 ± 9.7
LM.0	340.4 ± 2.5	356.1 ± 9.7	407 ± 15.3	449.4 ± 6.4	524.9 ± 7.6	539.4 ± 8.4	562.8 ± 3.9	575.1 ± 2.6	592.2 ± 6.8	607.6 ± 12.5	632.5 ± 8.5
SM.4	419.5 ± 3.4	453.7 ± 6.8	506.8 ± 10.9	527.3 ± 4.4	587.4 ± 6	604.3 ± 6.2	596.8 ± 9.7	641.8 ± 6.4	672.8 ± 5.7	696.6 ± 7.7	702.1 ± 6.8
LM.4	342.5 ± 2.2	375.9 ± 3.5	417.2 ± 9.7	443.9 ± 6.7	501.4 ± 11.5	533.9 ± 6.8	579.4 ± 10.1	635.2 ± 3.7	654.1 ± 14.7	672.6 ± 11.2	696.9 ± 7.2
SM.10	1480 ± 6.7	2456 ± 8.8	2681.2 ± 5.9	3015 ± 8.0	3517 ± 14.1	3764.3 ± 9.7	3907.1 ± 11.6	4061 ± 10.5	4197 ± 11.2	4297.7 ± 8.1	4363 ± 11.2
LM.10	390 ± 9.5	415.3 ± 6.7	426.6 ± 6.7	459.9 ± 11.6	599.5 ± 15.1	644.7 ± 11.5	704 ± 10.7	722.2 ± 11.7	760.1 ± 8.7	827.4 ± 9.3	921.8 ± 9.7
SM.24	458.2 ± 8.7	520.7 ± 9.1	572.7 ± 2.5	605.4 ± 15.9	689.8 ± 10.3	696.9 ± 8.6	752.4 ± 9.2	827.9 ± 8.1	852.9 ± 7.8	873.1 ± 7.4	889.5 ± 3.9
LM.24	368.6 ± 2.6	382.5 ± 11.6	413.7 ± 6.4	457.3 ± 13.4	527.1 ± 9.7	571.09 ± 6.7	628.4 ± 8.9	668.6 ± 7.8	694.6 ± 11.3	723.9 ± 6.3	752.8 ± 4.1

Results (displayed as mean values ±SD, n = 3), SM – storage modulus, LM – loss modulus.

Supplementary Table 4. Standard deviation of storage (G′) and loss (G′′) shear moduli for 95% wheat + 5% soy flour with *Lp*.

	**0.628**	**5**	**13**	**20**	**45**	**68**	**151**	**227**	**341**	**513**	**628**
SM.0	733.2 ± 8.4	929.4 ± 4.6	1068.8 ± 9.4	1134.5 ± 7.1	1249.2 ± 3	1342.1 ± 8.6	1519.6 ± 9.4	1698.9 ± 6.1	1957.4 ± 2.3	2525.4 ± 4.1	2493.3 ± 5.7
LM.0	354.4 ± 6.2	400.8 ± 6.7	474.9 ± 8.9	510.8 ± 10.5	595.7 ± 3.4	645.5 ± 10.4	794.5 ± 7.5	878.2 ± 12.4	937.9 ± 13.1	1131.1 ± 14.5	1447.3 ± 4.1
SM.4	882.1 ± 3.9	1186.5 ± 8.9	1343.3 ± 9.3	1420.4 ± 7.4	1561.9 ± 9.1	1674 ± 11.6	1936.3 ± 6.4	2154.3 ± 7.5	2516.5 ± 3.8	3298.6 ± 15.2	3854.2 ± 9.2
LM.4	419.7 ± 11.5	501.4 ± 11.5	580.7 ± 10.5	617.2 ± 6.2	702.6 ± 7.4	756.1 ± 9.7	906.3 ± 7.9	991.9 ± 2.8	1065.5 ± 9.7	1202.6 ± 9.9	1527.9 ± 8.4
SM.10	1551.7 ± 6.7	1880.6 ± 16.7	2026.4 ± 4.7	2189.3 ± 3.7	2293 ± 9.2	2376.4 ± 8.8	2483.6 ± 8.1	2550.7 ± 11	2578 ± 12.2	2585.9 ± 7.8	2609.5 ± 7.5
LM.10	581.2 ± 7.8	680.1 ± 9.7	721.8 ± 5.6	778.9 ± 10.8	820.8 ± 7.8	896.3 ± 6.4	973.2 ± 11.4	1050.7 ± 9.7	1092.3 ± 9.8	1143.2 ± 6.8	1191 ± 6.4
SM.24	1093.2 ± 10.3	1202.7 ± 8.4	1380.4 ± 8.6	1462.5 ± 9.4	1528.8 ± 6	1595.9 ± 6.7	1651.6 ± 3.8	1699.1 ± 4.6	1734.5 ± 7.1	1797.1 ± 9.4	1816.4 ± 8.2
LM.24	455.4 ± 7.2	476.2 ± 2.2	564.5 ± 3.4	590.4 ± 9.4	617.7 ± 8.3	650.9 ± 4.8	709.8 ± 6.4	737.3 ± 5.7	759.8 ± 4.8	820.6 ± 11	894.3 ± 7.5

Results (displayed as mean values ±SD, n = 3), SM – storage modulus, LM – loss modulus.

Supplementary Table 5. Standard deviation of storage (G′) and loss (G′′) shear moduli for 95% wheat + 5% soy flour with *Lc*.

	**0.628**	**5**	**13**	**20**	**45**	**68**	**151**	**227**	**341**	**513**	**628**
SM.0	1247 ± 8.1	1433.4 ± 1.3	1564.2 ± 5.7	1601.2 ± 4.9	1620.2 ± 4.5	1744.2 ± 8.4	1805.8 ± 2.3	1860.5 ± 6.7	1959.4 ± 2	2051.1 ± 3.8	2081.4 ± 6.2
LM.0	456.7 ± 7.6	504.4 ± 8.6	560.4 ± 3.2	569.2 ± 7.4	623.1 ± 6.3	660.4 ± 6.3	719.4 ± 1.9	767.1 ± 1.5	850.8 ± 7.7	944.5 ± 4.2	1033.8 ± 7.3
SM.4	1502 ± 8.9	1941.7 ± 9.7	2163.6 ± 3.6	2274.7 ± 6.7	2532.3 ± 9.5	2661.5 ± 7.9	2986.6 ± 1.8	3153.3 ± 9.4	3224.2 ± 3.7	3277 ± 11.5	3366.8 ± 9.2
LM.4	570.5 ± 11.5	634 ± 2.6	735.4 ± 8.1	788.9 ± 4.6	927.9 ± 8.9	1030.1 ± 8.4	1237.4 ± 5.6	1375.2 ± 3.7	1454.5 ± 8	1524.3 ± 3.7	1649.4 ± 7.4
SM.10	1444.6 ± 9.3	1901.8 ± 8.4	2008.7 ± 8.4	2136.7 ± 7.5	2274.8 ± 9.2	2376.8 ± 9	2503.9 ± 6.9	2612.9 ± 14.1	2738.8 ± 4.6	2777.9 ± 4.6	2803.2 ± 9.7
LM.10	588.9 ± 4.4	656.7 ± 7.1	705.1 ± 11.6	746.5 ± 11	834 ± 13.5	882 ± 5.6	959.1 ± 9.4	1060.1 ± 3.7	1159.4 ± 5.7	1209.5 ± 9.4	1268.7 ± 2.3
SM.24	1176.9 ± 6.7	1414.6 ± 11	1670.4 ± 13	1771.4 ± 2.1	1877.5 ± 3.7	1964.7 ± 4.3	2056 ± 14.2	2182 ± 3.9	2221.7 ± 6.2	2243.2 ± 15	2255.3 ± 14.9
LM.24	503.8 ± 7.1	518.9 ± 6.9	662.4 ± 7.7	716.7 ± 4.5	776.9 ± 9.3	853.2 ± 2.7	962.2 ± 2.1	1059.6 ± 5.6	1122.4 ± 7.6	1187.7 ± 9.7	1258.5 ± 8.5

Results (displayed as mean values ±SD, n = 3), SM – storage modulus, LM – loss modulus.

Supplementary Table 6. Standard deviation of storage (G′) and loss (G′′) shear moduli for 95% wheat + 5% soy flour with *Lp* + *Lc* + *Sc***.**

	**0.628**	**5**	**13**	**20**	**45**	**68**	**151**	**227**	**341**	**513**	**628**
SM.0	1150 ± 16.1	1349.3 ± 4.5	1484.8 ± 5.6	1569.2 ± 4.7	1657.9 ± 8.9	1730.1 ± 5.5	1806.3 ± 3.4	1867.9 ± 7.4	1983.4 ± 4.7	2020.1 ± 8.8	2048.5 ± 11.4
LM.0	517 ± 8.9	573.7 ± 6.3	634.3 ± 15.3	663.9 ± 6.6	703.1 ± 9.7	743.9 ± 6.8	792.1 ± 8.9	875.9 ± 6.1	939.5 ± 10.3	976.8 ± 4.6	1018.6 ± 8.5
SM.4	1823.9 ± 6.7	2094 ± 11.8	2241.3 ± 13.2	2480.9 ± 5.9	2602.1 ± 6.3	2790.3 ± 4.9	2925.3 ± 6.7	3042.3 ± 3.6	3210.5 ± 9.4	3265.8 ± 1.4	3322.5 ± 3.5
LM.4	701.9 ± 7.6	751.8 ± 7.4	801 ± 10.1	902 ± 11	951.8 ± 4.7	1037.7 ± 6.6	1120.2 ± 4.7	1234.2 ± 5.1	1339 ± 12.6	1396.9 ± 2.4	1541.1 ± 6.8
SM.10	1495.6 ± 4.7	1784 ± 3.9	2056.9 ± 12.1	2236.8 ± 9.4	2456.7 ± 9.5	2656.1 ± 3.8	2743.8 ± 9.5	2831.7 ± 6.4	2907.2 ± 8.8	3027.6 ± 8.9	3103.6 ± 4.5
LM.10	658.6 ± 7.6	696.5 ± 2.5	809.4 ± 8.8	883.7 ± 6.1	1016.9 ± 8.1	1154.9 ± 8.1	1209.6 ± 7.5	1263.6 ± 5.1	1320.4 ± 9.4	1462.8 ± 4.6	1617.7 ± 4.3
SM.24	845.7 ± 8.8	977.6 ± 6.2	1064.6 ± 6.6	1110.8 ± 8.7	1214.2 ± 7.6	1280.3 ± 8.1	1322.8 ± 8.9	1391 ± 12.1	1468.7 ± 6.7	1489.8 ± 11.5	1496.7 ± 9.5
LM.24	528.5 ± 12.2	561.5 ± 1.9	574.7 ± 5.7	631.6 ± 6.3	651.9 ± 11.2	675.9 ± 12.9	698.9 ± 5.3	802.3 ± 8.4	844.2 ± 10.3	874 ± 8.7	904 ± 3.2

Results (displayed as mean values ±SD, n = 3), SM – storage modulus, LM – loss modulus.

Supplementary Table 7. Standard deviation of storage (G′) and loss (G′′) shear moduli for 90% wheat + 10% soy flour with *Lp*.

	**0.628**	**5**	**13**	**20**	**45**	**68**	**151**	**227**	**341**	**513**	**628**
SM.0	1196.6 ± 8.4	1365.8 ± 9.4	1471.6 ± 5.7	1542.1 ± 3.7	1690.2 ± 9.4	1812.3 ± 7.4	1990.4 ± 2.3	2093.3 ± 4.7	2127.1 ± 1.4	2192.3 ± 8.5	2226.9 ± 7.4
LM.0	547.7 ± 9.8	547.6 ± 8.6	561.3 ± 2.2	600.3 ± 11.4	626.6 ± 10.5	654.6 ± 3.7	758.7 ± 10.3	810.5 ± 5.8	846.9 ± 13.2	910.7 ± 3.2	989.7 ± 9.1
SM.4	1002.2 ± 4.8	1103.1 ± 5.3	1139.3 ± 9.8	1244.6 ± 5.4	1325.1 ± 6.8	1421.9 ± 9.9	1517.1 ± 6.9	1591.8 ± 12	1761.3 ± 5.6	1827.1 ± 8.9	1868.9 ± 7.9
LM.4	472.2 ± 4	479.5 ± 10.2	505.6 ± 6.7	538.8 ± 9.1	545.7 ± 6.7	598.6 ± 8.4	638 ± 3.8	713.8 ± 11.3	811.7 ± 2.5	877.8 ± 5.5	945.1 ± 11.1
SM.10	608.6 ± 6.1	622 ± 8.7	690.9 ± 5.6	749.2 ± 7.2	786.8 ± 8.4	871.4 ± 8.2	921.8 ± 6.4	971.9 ± 3.3	1074.3 ± 3.8	1155.7 ± 14	1203.9 ± 5.3
LM.10	408.8 ± 9.9	368.6 ± 6.6	377.1 ± 8.4	399.3 ± 8.4	459.4 ± 9.7	482.1 ± 9.2	520.5 ± 9.4	592.4 ± 2.6	648.5 ± 11.2	728.5 ± 6.7	837 ± 8.4
SM.24	1909.7 ± 11	2265.1 ± 7.5	2447.5 ± 4.4	2503.1 ± 3.6	2654.7 ± 5.6	2788.5 ± 5.2	2916.9 ± 7.3	3047.5 ± 8.2	3149.9 ± 7.8	3273.4 ± 9.4	3313.6 ± 6.7
LM.24	696.5 ± 12.3	739.9 ± 7.9	798.8 ± 4.8	802.9 ± 7.4	858.3 ± 10.4	916.4 ± 7.8	955.6 ± 5.1	1027.6 ± 7.1	1116.7 ± 3.9	1205.8 ± 9.1	1314.6 ± 4.7

Results (displayed as mean values ±SD, n = 3), SM – storage modulus, LM – loss modulus.

Supplementary Table 8. Standard deviation of storage (G′) and loss (G′′) shear moduli for 90% wheat + 10% soy flour with *Lc*.

	**0.628**	**5**	**13**	**20**	**45**	**68**	**151**	**227**	**341**	**513**	**628**
SM.0	791.4 ± 2.2	903.4 ± 11.3	956.9 ± 8.8	1037.2 ± 2.2	1136.5 ± 8.5	1187.4 ± 6.8	1239.1 ± 7.7	1261 ± 5.7	1319.8 ± 5.6	1373.2 ± 5.8	1371.7 ± 8.7
LM.0	424.8 ± 12.3	419.9 ± 5.6	453.9 ± 4.7	482.1 ± 11.3	530 ± 6.4	553.4 ± 2.8	577.2 ± 7.4	626.4 ± 9.1	689.1 ± 9.4	752.8 ± 4.7	835.3 ± 4.9
SM.4	990.4 ± 9.8	1294.8 ± 7.7	1438.4 ± 5.6	1525.6 ± 6.1	1700.6 ± 9.3	1807.3 ± 1.7	2077.7 ± 3.1	2209 ± 8.3	2312.2 ± 6.3	2414.9 ± 3.9	2502.7 ± 6.1
LM.4	450.6 ± 5.3	548.9 ± 9.8	619.6 ± 7.1	674.2 ± 6.3	813.8 ± 4.4	897.4 ± 9.1	1106.7 ± 6.5	1252.9 ± 8.1	1385.8 ± 7.4	1542.6 ± 6.8	1726.5 ± 5.2
SM.10	890 ± 4.9	983.7 ± 8.7	1064.9 ± 4.8	1101.4 ± 3.8	1169.7 ± 8.5	1298.2 ± 14	1358.5 ± 7.4	1399 ± 6.5	1484.8 ± 5.4	1505.2 ± 5.9	1532.5 ± 2.9
LM.10	388.9 ± 6.8	410.4 ± 7.8	444.2 ± 8.2	479.6 ± 5.3	562.4 ± 4.9	594.3 ± 6.8	650.7 ± 7.8	701.3 ± 5.8	782.2 ± 6.5	838.5 ± 2.7	933.8 ± 11.4
SM.24	1684 ± 1.7	2155.1 ± 6.6	2320.2 ± 11	2538.7 ± 3.9	2775.3 ± 3.3	3414.2 ± 4.5	3846.7 ± 7.1	4953.9 ± 3.7	5105.2 ± 11.5	5400.8 ± 7.9	5790.3 ± 5.8
LM.24	820.4 ± 9.2	931.2 ± 10.4	994.7 ± 10.9	1058.8 ± 7.3	1140.8 ± 5.6	1337.3 ± 9.4	1520.1 ± 9	2015.7 ± 11	2167.5 ± 8.4	2347 ± 3.6	2614.3 ± 6.4

Results (displayed as mean values ±SD, n = 3), SM – storage modulus, LM – loss modulus.

Supplementary Table 9. Standard deviation of storage (G′) and loss (G′′) shear moduli for 90% wheat + 10% soy flour with *Lp* + *Lc* + *Sc*.

	**0.628**	**5**	**13**	**20**	**45**	**68**	**151**	**227**	**341**	**513**	**628**
SM.0	1221.6 ± 8.9	1420 ± 3.1	1520.1 ± 4.2	1604.4 ± 8.8	1727.2 ± 8	1900.5 ± 7.2	2051 ± 9.4	2116.3 ± 6.1	2226.8 ± 7.1	2399.1 ± 8.7	2456.1 ± 10.2
LM.0	661.3 ± 8.4	677.1 ± 3.8	694.5 ± 5.3	729.1 ± 9.4	769 ± 14.7	862.8 ± 14.1	929.4 ± 6.8	994.4 ± 3.6	1077.8 ± 8.4	1201.8 ± 9.2	1351.8 ± 6.7
SM.4	2167 ± 5.5	2396.5 ± 7.9	2572.9 ± 6.2	2787.5 ± 1.4	2960.3 ± 5.2	3192.4 ± 9.2	3430.9 ± 10.5	3637.2 ± 7.2	3933.2 ± 6.2	4170.3 ± 11	4324.2 ± 4.8
LM.4	886.7 ± 10.4	912.8 ± 9.6	923.6 ± 7.5	972.6 ± 3.7	1037.9 ± 9.1	1100.4 ± 3.3	1174.4 ± 11.8	1302.3 ± 3.4	1447 ± 6.7	1639.3 ± 9.4	1855.8 ± 8.4
SM.10	2407.6 ± 8.9	2674 ± 10.8	2903.9 ± 8.7	3123 ± 3.4	3385.8 ± 7.5	3640.3 ± 9.8	3936.8 ± 9.4	4252.9 ± 9.1	4595.1 ± 7.4	5071.5 ± 4.9	5749.9 ± 8.7
LM.10	916.6 ± 4.6	946.9 ± 7.5	1018.6 ± 3.8	1073.5 ± 4	1140.6 ± 2.2	1228.3 ± 5.8	1346.4 ± 5.8	1480.2 ± 10.4	1697.1 ± 8.6	1925.5 ± 5.4	2209.5 ± 11.5
SM.24	2917.2 ± 2.4	3179.1 ± 5.8	3433.2 ± 4.2	3703.1 ± 12	3963.7 ± 9.3	4350.8 ± 4.4	4557.8 ± 6.6	4763.1 ± 8.9	4947.8 ± 5.3	5169.7 ± 10	5326 ± 10.1
LM.24	990.8 ± 9.4	1032.2 ± 8.8	1081.7 ± 6.2	1122 ± 4.2	1206.1 ± 6.7	1307.8 ± 8.9	1370.5 ± 14.3	1468.6 ± 11.2	1581.3 ± 9.1	1725.6 ± 2.5	1888.6 ± 7.6

Results (displayed as mean values ±SD, n = 3), SM – storage modulus, LM – loss modulus.
